# Force Feedback Method for Statically Indeterminate Steel Structure Construction Based on Staged Temperature Measurement

**DOI:** 10.3390/s24248073

**Published:** 2024-12-18

**Authors:** Wei Lu, Xianwei Fang, Liming Liu, Chunfeng Ao, Weihua Hu, Jun Teng, Zhongcheng Huo

**Affiliations:** 1 School of Civil and Environmental Engineering, Harbin Institute of Technology (Shenzhen), Shenzhen 518000, China; xiaolidu11@163.com (X.F.); liulm0102@163.com (L.L.); huweihua@hit.edu.cn (W.H.); tengj@hit.edu.cn (J.T.); 2 China Construction Steel Engineer Co., Ltd., Shenzhen 518118, China; zjgg_aocf2017@cscec.com (C.A.); zjgg_huozc@cscec.com (Z.H.)

**Keywords:** statically indeterminate structure, thermal effect, construction monitoring, construction force feedback, construction safety

## Abstract

Structural design usually adopts uniform temperature action. However, during the actual construction of the structure, the temperature field acting on the structure is inhomogeneous. Therefore, the simulation of the construction of statically indeterminate steel structures considering only the uniform temperature field cannot truly reflect the temperature action after structural molding and the evolution of the stress performance of the temporary stress system of structural construction. This paper proposes a force feedback method for the construction of statically indeterminate steel structures based on staged temperature measurements. In this method, the construction sequence and the measured temperature are taken into account, and the structural response under the action of temperature is obtained by using the first processing method in the matrix displacement method and the deformation coordination formula to obtain the deformation and stress values of the structure. The application results show that the structural forces calculated by the method proposed in this paper are closer to the measured data in the field, and the error is reduced by 10~40%. This paper provides a reference for the calculation of construction forces in statically indeterminate steel structures considering the effect of temperature inhomogeneity and provides a basis for construction safety assessment and schedule management.

## 1. Introduction

Structural construction is formed by multiple construction temporary stress systems. The overall structural analysis at the design stage cannot adequately reflect the evolution of the structural stress performance during the construction process, while the structural construction simulation analyzes the stress performance of each construction temporary stress system according to the predefined construction steps. Aiming at the construction difficulty that the geometrical boundary conditions of the structure change over time during the construction process, the existing study proposes a construction simulation method that solves the “life and death” substructures independently and makes the results of the construction simulation closer to the prototype by simulating the force system of the different subsections separately [1]. In addition, there is a study for a large-span steel structure corridor using Midas software 2024 (v2.2) to simulate the construction steps of different sections of the structure, resulting in the use of the “two sides of the segmental lifting + middle of the whole lifting” program of the structural deformation, and component stress change is small [2]. It can be seen that a zoned segmental construction simulation method is more realistic for statically indeterminate steel structures of higher complexity. However, these studies were limited to considering only the constant and live load effects on the structure and did not consider the temperature effects during actual construction.

During the staged construction of the Suzhou Railway Station Renovation Project, seasonal temperature variations had a significant impact on the structural internal forces. Temperature action is a major controlling factor in the design of super-long-span steel structures [3]. In a study of large-span steel structures with large outdoor elements or large translucent roofs, it was found that non-uniform time-varying temperature effects occur on the surface of the structure under the action of intense solar radiation, causing large temperature stresses and deformations in the structure [4]. Numerical simulation of the main truss lifting, the structural installation process, and the support unloading process using different unloading methods for the large-space steel structure roof cover of Tianjin Olympic Center Stadium found that sunshine would lead to an uneven temperature distribution on the surface of the structural rods, which would result in large temperature stresses on the rods [5]. It can be seen that the temperature change has an important influence on the formation of the temporary stress system during the construction of the statically indeterminate steel structure and the structural stress performance after the construction is completed. Therefore, it is necessary to consider the effect of non-uniform temperature variation on the actual forces in the construction of statically indeterminate steel structures to provide a theoretical basis for structural construction safety and design optimization.

Due to the long construction period of the statically indeterminate steel structure and the more complex structural form, the structure is subjected to a non-uniform temperature field that varies with the construction period [6,7,8,9], which presents the characteristics of time-varying structures, time-varying material, and time-varying boundary conditions [10,11,12]. Both the structure formation path and the temperature effects directly affect the stress performance of the structure during the construction phase and the service phase. Current research on the role of temperature during the construction phase of structures focuses on methods for determining the construction closing temperature of large-span, statically indeterminate steel structures. A study has utilized the finite unit method to calculate the temperature stresses of the closing members at different time periods so as to determine the appropriate closing time periods and corresponding closing temperatures for the stadium structure and to provide a reference for the closing construction of large-span space steel structures [13]. A study has used finite element analysis software to calculate the temperature stresses of single-story, super-long frame structures at different closing times under the premise of considering the internal forces in each construction stage of the construction process [14]. A study has investigated the effect of temperature on the construction process from three aspects: the overall structure, the zoning construction steps, and the structural closure, and the calculation results show that setting different temperature zones and adopting different zoning closure sequences have an important effect on the internal force during the construction and molding of the overall structure [15]. It can be seen that the current simulation analysis of the construction of statically indeterminate steel structures is mostly used for the determination of closing time, closing temperature, and closing sequence. It is necessary to further investigate the feedback of the construction stress state under the action of non-uniform temperature, to consider the influence of the initial temperature on the structural construction stress performance of different structural components at the time of installation in stages, and to use the on-site monitoring of the temperature to update the construction simulation results in real-time, so as to realize the tracking of the structural stress state in synchronization with the construction and the feedback.

The current research on construction monitoring of super statically indeterminate steel structures mainly considers the force and deformation performance of super statically indeterminate steel structure construction under constant and live loads. By simulating and analyzing the forces and deformations of a gymnasium in Xi’an at eight different construction stages under constant and live load conditions, the results were compared with the stress monitoring data to predict the unfavorable construction nodes and ensure the safety of structural construction [16]. During the construction process of the Hefei Binhu International Convention and Exhibition Center project, through the real-time stress monitoring of key nodes and the use of finite element software for the simulation of key node stress, they obtained the simulated value of the key node stress and the monitoring value and made a comparison to be used in the later construction process of the project to provide guidance on the safety of the project [17]. It can be seen that the current construction site monitoring program mainly monitors the critical member stresses and uses the simulated stresses under constant load and live load conditions as the early warning values, ignoring the non-uniform temperature effects that exist during the construction process. Therefore, it is necessary to consider the effect of non-uniform temperature action in structural construction phasing for better feedback and safety assessment of structural forces during construction.

In this paper, we propose a force feedback method for the construction of statically indeterminate steel structures based on staged temperature measurements, in which the temperature monitoring data at the time of component installation are used as the initial temperature. Based on the formation process of the statically indeterminate structural system, the construction structure group is divided, and the construction simulation method based on the staged initial temperature measurement is established. Real-time tracking of the stress state of the temporary stress system occurs at each stage of construction through construction simulation, comparing the component stress monitoring values with the component stresses obtained by the proposed construction simulation method, and verifying the validity of the construction simulation method. Further feedback is provided on the structural construction stress based on the construction model and the measured temperature role of real-time access to the component stress distribution of the molded temporary stress system.

This method utilizes temperature sensors to implement tracking monitoring of component temperature and imposes a measured non-uniform temperature field on each construction temporary stress system in the actual construction process according to the real temperature data obtained, which reduces to a certain extent the adverse effects of ignoring the non-uniform temperature effects in the construction process on the safety of structural construction. The proposed method was applied in the construction monitoring of the Shenzhen Nanshan Science and Technology Innovation Center, and the effectiveness and feasibility of the method were verified.

The main contribution of this paper is to consider the effect of non-uniform temperature field action on structural construction during the construction process of a structure and to propose a force feedback method for the construction of statically indeterminate steel structures based on staged temperature measurements. This will generate a more realistic component stress distribution of the formed temporary stress system in real-time using the construction model and the role of the measured temperature so as to provide feedback on the structural construction force for the safety of structural construction and the pre-adjustment of the construction program to provide a scientific basis.

The rest of the paper is organized as follows: Section 2 introduces the research methodology, which details the proposed statically indeterminate steel structure construction force feedback method based on staged temperature measurements from the three parts of the construction structure group division, the determination of the temperature action value, and the construction force feedback. It also analyzes the construction scheme and the difficulties of the Science and Technology Innovation Center project in Nanshan, Shenzhen. Section 3 determines the construction plan of the Nanshan Science and Technology Innovation Center project and carries out the tracking measurement of the temperature, divides the structural group, and determines the temperature working condition according to the method proposed in this paper. Section 4 compares and analyzes the monitored and simulated values of structural displacements and stresses during the construction of the project, respectively. Section 5 draws some conclusions and makes recommendations for future research.

## 2. Methods

### 2.1. Force Feedback Method for Statically Indeterminate Steel Structure Construction Based on Staged Temperature Measurement

#### 2.1.1. Modeling of the Construction Steps and Construction Structure Grouping Principles

The building structure in a construction process requires considering the construction progress and time schedule to refine the entire construction process and considering the effect of the division of construction steps on the smooth progress of a construction project and the final quality of the structure. Owing to the complexity of the steps in a real construction process, the finite element software used for simulating the construction process may not comply with the construction process of each step of the rod installation process in the construction of the structural group. Therefore, at this stage of the construction simulation, multiple adjacent rods are divided into a construction structural group; however, this ignores the splicing process of the rods in the formation of the statically indeterminate structural system of the formation of the order when subjected to the action of temperature, structural simulation stress performance, and actual state when there is a certain gap. Furthermore, there is a gap between the simulated stress performance of the structure and the actual state when subjected to temperature effects, and therefore, when dividing the structural group, we need to consider the construction sequence of the statically indeterminate structural system to ensure that the results of the construction simulation are closer to the actual construction structure.

During the construction of building structures, the overall construction steps are decided by the construction unit and summarized in the construction plan. The construction process simulation is completed before construction, and therefore, it cannot accurately reflect the formation sequence and stress changes in the rods during the construction process. The construction force feedback method proposed in this study tracks the construction process while determining the assembly sequence and installation temperature of the bars more accurately to obtain a more accurate temperature action. Meanwhile, for a statically indeterminate space structure, the temperature effect is an important factor that affects the deformation of the structural force, and therefore, it is very important to consider the construction structure group division of the statically indeterminate structure formation sequence in the process of structural construction simulation.

For a statically indeterminate structure, temperature action is an important factor that affects the stresses on the structure; when the temperature of the statically indeterminate structure changes, the statically indeterminate structure will generate temperature stresses. For general statically indeterminate structures, the force method is usually used for the calculation of temperature action. The calculation process starts by lifting the redundant constraints and replacing them with basic unknowns, which can be forces or moments. As an example, the nth statically indeterminate constraint can be replaced by the basic unknown quantity A, which can be expressed as follows:

(1) $$\bar{X}=[X_{1}\;X_{2}\;\ldots \;X_{i}\ldots \;X_{n}]$$

where $\bar{X}$ represents the overall basic unknown, and $X_{i}$ represents the *i*th basic unknown.

The overall structural deformation matrix can be expressed as follows:

(2) $${\bar{\Delta }}_{t}=[\Delta_{1t}\;\Delta_{2t}\;\ldots \;\Delta_{it}\ldots \;\Delta_{nt}]$$

where ${\bar{\Delta }}_{t}$ represents the deformation due to the fundamental unknown $\bar{X}$, and $\Delta_{it}$ represents the deformation due to the unit force in the direction of the *i*th unknown.

For the *i*th deformation, the computational procedure can be expressed as follows:

(3) $$\Delta_{it}=\sum \left(\pm \right)\alpha t_{0}\omega_{\bar{N}}+\sum (\pm )\frac{\alpha \Delta_{t}}{h}\omega_{\bar{M}}$$

where α represents the coefficient of linear expansion, $t_{0}$ represents the average change in temperature, $\omega_{\bar{N}}$ represents the result of plot multiplication of axial force per unit force, $\Delta_{t}$ represents the temperature difference, h represents the height of the bar, and $\omega_{\bar{M}}$ represents the result of plot multiplication of bending moment per unit force.

(4) $$\delta_{i1}X_{1}+\delta_{i2}X_{2}+\ldots +\delta_{ij}X_{j}+\ldots +\delta_{in}X_{n}+\Delta_{it}=0$$

where $\delta_{ij}$ represents the deformation induced by the unit force *j* in the *i*-direction, $X_{j}$ represents the equivalent alternative force in the *j*-direction, and $\Delta_{it}$ represents the deformation induced by the temperature action in the *i*-direction.

The positive and negative signs are specified in the following manner: For axial forces, the tensile force is defined as a positive sign, and for temperature changes, the temperature increase is also defined as a positive sign. For the product of bending moments and temperature differences, if they cause tensile deformations on the same side of the bar, the product is positive; otherwise, it is negative. After calculating the displacement, the overall bending moment of the structure can be calculated using the bending moment superposition formula, which can be expressed as follows:

(5) $$M={\bar{M}}_{1}X_{1}+{\bar{M}}_{2}X_{2}+\ldots +{\bar{M}}_{j}X_{j}+\ldots +{\bar{M}}_{n}X_{n}$$

where M represents the overall bending moment, ${\bar{M}}_{j}$ represents the bending moment produced when the unit force $X_{j}=1$ acts alone, and $X_{j}$ represents the equivalent replacement force in the j direction.

After deriving the structural bending moments, the structural stresses can be obtained using the stress solution equation, which can be expressed as:

(6) $$\sigma =\frac{M\cdot y}{I_{z}}$$

where σ represents the positive stress, M represents the overall bending moment, y represents the distance from the neutral axis, and $I_{z}$ represents the moment of inertia of the cross-section against the z-axis.

In this study, we consider the construction steps and the formation sequence of the statically indeterminate structural system and structural components constructed at the same stage. Furthermore, the part that can form a statically indeterminate system with the structure is divided into a structural group, assuming that a structure can be divided into m construction structural groups during the construction process. Finally, the overall construction structural group matrix is expressed as follows:

(7) $$\bar{J}=[J_{1}\;J_{2}\;\ldots \;J_{i}\ldots \;J_{m}]$$

where $\bar{J}$ and $J_{i}$ represent the overall structural group matrix and the *i*th structural group, respectively.

#### 2.1.2. Determination of the Value of Temperature Action Based on Staged Measurements

A longer period of time is required during the closing process in the construction phase because the form of statically indeterminate space structures is more complex, during which overall stresses on the structure and the process of coordinating the deformation of the structure become more complex. In this case, special attention needs to be paid to the effects of temperature action during design and construction to ensure the safety and stability of the structure. Based on the principles of construction structure grouping proposed in Section 2.1, a finer distinction is made between the initial temperatures of the bars within a single construction phase group. For an *i*th construction structure group $J_{i}$, assuming that the number of its rods is n, the initial temperature matrix of the structure group $J_{i}$ based on the initial temperature of each rod measured by the temperature stress sensors installed on the structure can be expressed as follows:

(8) $$T_{i}=[T_{10}\;T_{20}\;\ldots \;T_{j0}\;\ldots \;T_{n0}]$$

where $T_{i}$ represents the initial temperature matrix of the *i*th structural group, and $T_{j0}$ represents the initial temperature of the *j*th rod.

Subsequently, during the construction of the structure, the overall statically indeterminate structural system changes constantly, the structure is subjected to a constant temperature effect, and the overall structural stress becomes extremely complex. The temperature magnitude of each component of the structure at this point can be obtained by the temperature monitoring sensor for a certain moment of the subsequent construction, e.g., the *i*th construction structure group $J_{i}$. The temperature magnitude at this point measured by the temperature monitoring sensor is expressed as follows:

(9) $$T_{it}=[T_{1t}\;T_{2t}\;\ldots \;T_{jt}\;\ldots \;T_{nt}]$$

where $T_{it}$ represents the matrix of the temperature monitoring values of structure group *i* at time t, and $T_{jt}$ represents the temperature monitoring value of the *j*th rod of structure group *i* at time *t*.

The magnitude of the temperature action for the *j*th structural group is obtained according to the following temperature action formula:

(10) $$\Delta T_{it}=[\Delta T_{1t}\;\Delta T_{2t}\;\ldots \;\Delta T_{jt}\;\ldots \;\Delta T_{nt}]$$

where $\Delta T_{it}$ represents the temperature action matrix of structure group *i* at time *t,* and $\Delta T_{jt}$ represents the temperature action of the *j*th rod of structure group *i*.

The specific calculation process of $\Delta T_{jt}$ is expressed as follows:

(11) $$\Delta T_{jt}=T_{jt}-T_{j0}$$

where $T_{j0}$ represents the initial temperature of the *j*th rod of structure group *i*, and $T_{jt}$ represents the temperature monitoring value of the *j*th rod of structure group *i* at time *t*.

#### 2.1.3. Construction Force Feedback Considering Staged Temperature Action

The stiffness matrix of the currently activated structure groups is denoted as $K_{1A}$, $K_{2A}$…$K_{jA}$ when the construction reaches the *j*th construction structure group, and the stiffness matrix of structure groups not mounted on the structure in the “kill” phase can be denoted as $K_{(j+1)D}$, $K_{(j+2)D}$…$K_{mD}$, where A represents the activated structure group and D represents the killed structure group.

The matrix displacement method is used to solve the force state of each structural group. The overall stiffness matrix is derived through the subunit stiffness matrix. The overall coordinate description of the unit stiffness matrix $K_{jA}$ is based on the displacement numbering of elements assembled into the structure of the overall stiffness matrix $K_{A}$. First, the nodes of the support, such as the zero displacement, are eliminated, and the structural stiffness matrix is a square matrix arranged in the displacement for determining the structure of the unknown displacement Δ (degrees of freedom).

Each element of the stiffness matrix is a nodal force under the action of the unit displacement of a node, which can be derived using the direct stiffness method. The total stiffness matrix of the structure K is formed directly by assembling the stiffness matrices of the units in overall coordinates.

The stiffness matrix formed by the prior processing method is characterized as symmetric, banded, sparse, and non-singular. The unitary stiffness matrix R of the rod is expressed as follows:

(12) $$R=\left[\begin{array}{cccccc} \frac{EA}{l} & 0 & 0 & -\frac{EA}{l} & 0 & 0\\ 0 & \frac{12EI}{l^{3}} & \frac{6EI}{l^{2}} & 0 & -\frac{12EI}{l^{3}} & \frac{6EI}{l^{2}}\\ 0 & \frac{6EI}{l^{2}} & \frac{4EI}{l} & 0 & -\frac{6EI}{l^{2}} & \frac{4EI}{l}\\ -\frac{EA}{l} & 0 & 0 & \frac{EA}{l} & 0 & 0\\ 0 & -\frac{12EI}{l^{3}} & -\frac{6EI}{l^{2}} & 0 & \frac{12EI}{l^{3}} & -\frac{6EI}{l^{2}}\\ 0 & \frac{6EI}{l^{2}} & \frac{4EI}{l} & 0 & -\frac{6EI}{l^{2}} & \frac{4EI}{l} \end{array}\right]$$

where EI represents the lateral stiffness, and l represents the unit length.

The unit stiffness matrix is transformed by the coordinate system transformation matrix T, and the overall stiffness matrix of all activated subunits can be obtained by assembling. The coordinate system transformation matrix T can be expressed as follows:

(13) $$T=\left[\begin{array}{cccccc} \cos\alpha & \sin\alpha & 0 & 0 & 0 & 0\\ -\sin\alpha & \cos\alpha & 0 & 0 & 0 & 0\\ 0 & 0 & 1 & 0 & 0 & 0\\ 0 & 0 & 0 & \cos\alpha & \sin\alpha & 0\\ 0 & 0 & 0 & -\sin\alpha & \cos\alpha & 0\\ 0 & 0 & 0 & 0 & 0 & 1 \end{array}\right]$$

where α represents the angle between the local and overall coordinate systems.

The overall stiffness matrix $K_{A}$ of the activated substructure and the unit stiffness matrix $K_{D}$ of the killed substructure are assembled using the above equations. Based on $K_{A}$ and $K_{D}$, and combined with the temperature action conditions, the structural response under temperature action can be calculated using the following deformation coordination formula:

(14) $$\left\{K_{A}+K_{D}\}\{u\}=\{\Delta T\right\}$$

where u represents the structural response under temperature action, and ΔT represents the temperature action load matrix.

Structural response A is used to obtain the overall structural response of the overall structure under the action of temperature, which is based on the structural response to obtain the deformation of the structure and magnitude of the stress, to obtain feedback on the construction process, and to guide the planning of the construction program. The stress situation of the structure during actual construction can be determined by carefully analyzing and evaluating the overall structural stress performance so that feedback can be provided on the overall structural stress and potential safety hazards and quality issues can be avoided.

### 2.2. Shenzhen Nanshan Science and Technology Innovation Center Project Profile

#### 2.2.1. Project Overview

**Figure 1:**
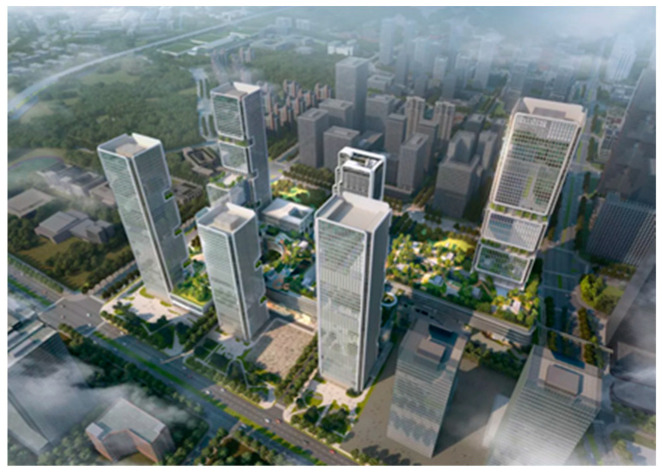
Nanshan Science and Technology Innovation Center.

The Shenzhen Nanshan Science and Technology Innovation Center is located in the core location of the headquarters base in Liuxiandong, Nanshan District, Shenzhen, with “urban ecological rainforest” as the design concept. It includes seven towers (160–250 m) as the “canopy”, with a huge super-static steel platform (~400 m long and 110 m wide) surrounding the middle of the 7th to 11th floors of the towers and constituting the “umbrella layer” of the rainforest. A rendered image is presented in Figure 1.

**Figure 2:**
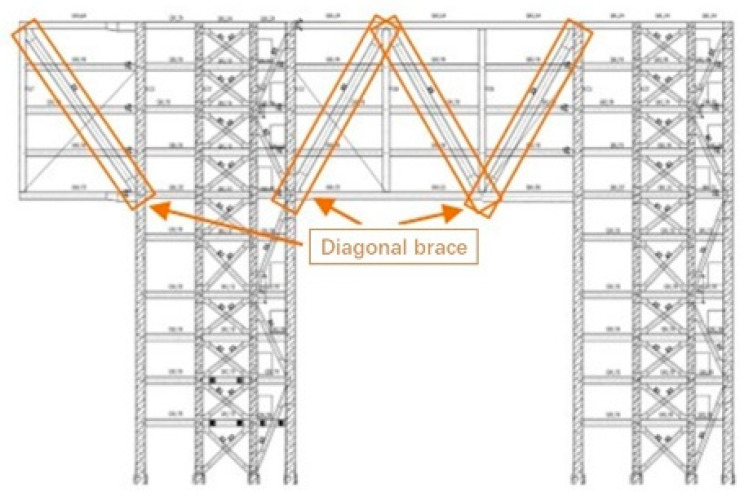
Nanshan Science and Technology Innovation Center truss diagram.

The main part of the monitoring project for the three buildings of the Nanshan Science and Technology Innovation Center is the steel structure of the large-span podium, which consists of tie beams and truss structures and is located on the 7th to 11th floors of Building 3. It is a 4-story, super-large, statically indeterminate steel platform with a width of ~90 m and a length of ~180 m, surrounded by a cantilevered structure. The single-bay truss structure is shown in Figure 2.

**Figure 3:**
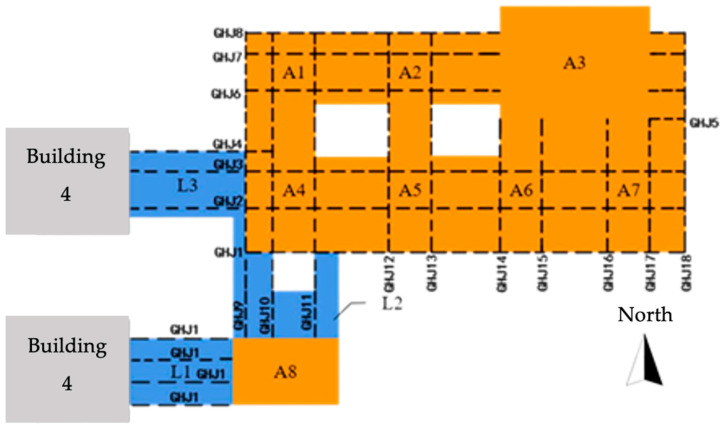
Schematic plan of the podium steel structure and connecting corridor.

The three buildings of the project contain a total of seven cores, which are denoted as cores A1–A7. The steel structure of the podium connects cores A1–A7 into a whole, and the maximum span between cores is 38 m, located between cores A1 and A4 and cores A2 and A5. The maximum span of the outer pick is 15 m, located on the south side of core cylinders A4, A5, A6, and A7. A schematic of the steel structure of the podium and connecting corridor is shown in Figure 3, where GHJ represents the steel truss number.

#### 2.2.2. Construction Program and Difficulties

**Figure 4:**
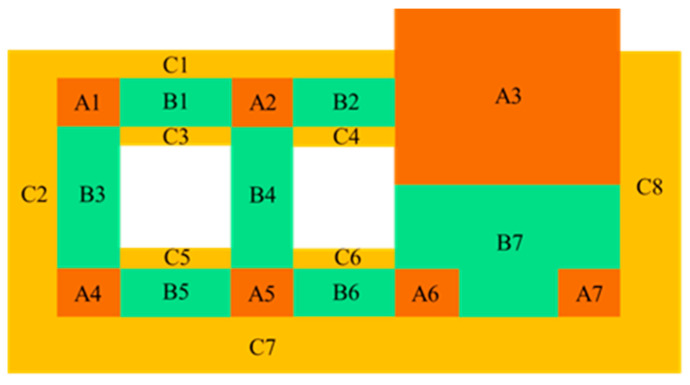
Construction structure division diagram.

**Figure 5:**
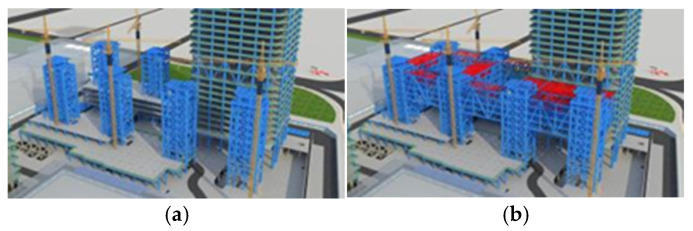

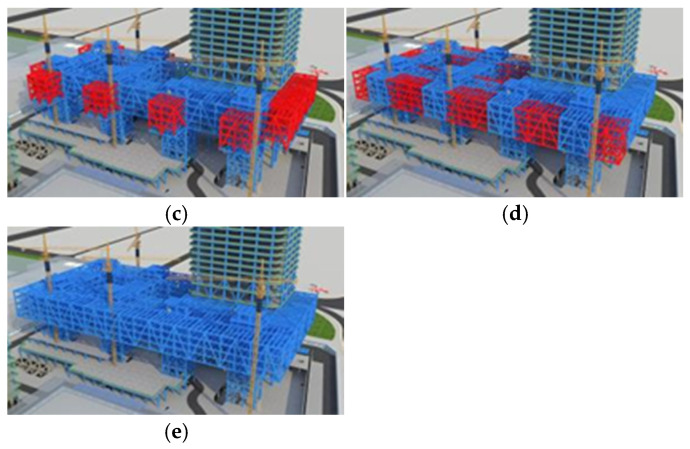
Overall construction flowchart: (**a**) Construction of A1–A7 completed; (**b**) B1–B7 large-span truss installation; (**c**) C1–C8 core connection suspension installation; (**d**) C1–C8 connecting corridor and corner installation; and (**e**) removal of diagonal bracing and complete unloading.

The construction project of the Nanshan Science and Technology Innovation Center was divided into three parts, as shown in Figure 4: A1–A7 for the core structure, B1–B7 for the large-span corridor structure, and C1–C8 for the outer cantilever structure. In Figure 4, areas B1–B7 are constructed using the construction program of inclined temporary support, areas C1–C8 are constructed using the overhead in situ bulk construction program, and the overall construction process is shown in Figure 5.

Difficulties in the construction process are as follows:

(1) The steel truss diagonal bracing of the podium goes through the 7–11th floors, as shown in Figure 2. There are many subsections of the components, the node form and stress condition are complicated, and the construction accuracy of the on-site installation must be high.
(2) Temporary supports are used during construction, and the structure undergoes complex force transformations during installation and unloading, making it difficult to assess the mechanical state.
(3) The construction period is long, the temperature change is obvious, and the structure belongs to statically indeterminate large-span continuous steel structures. Furthermore, there is a significant temperature effect.

## 3. Structural Construction Monitoring Program and Monitoring Data

### 3.1. Construction Monitoring Program

**Figure 6:**
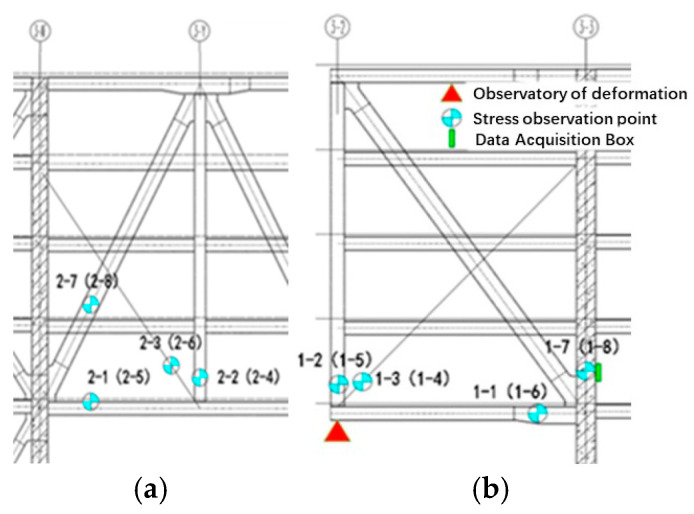
Layout of the measurement points of steel trusses (**a**) 2 and (**b**) 7.

**Figure 7:**
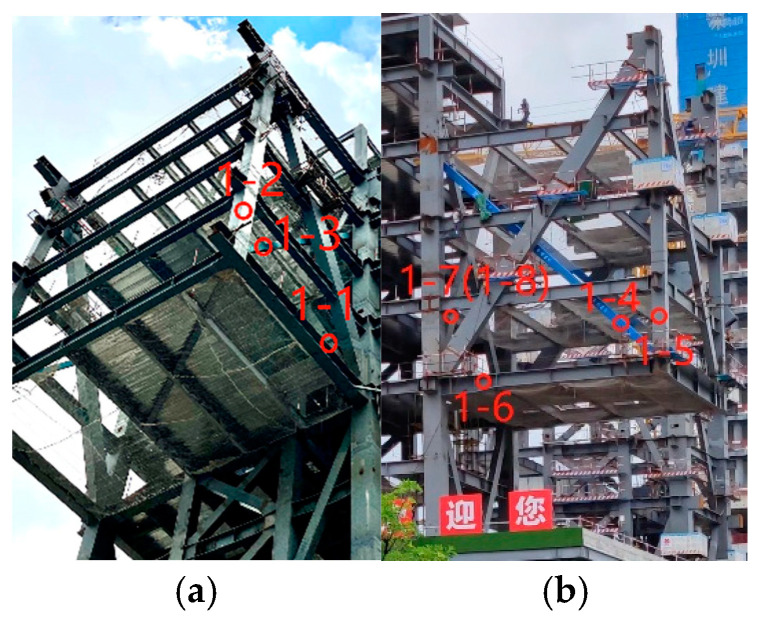
Field layout of the measurement points of steel truss 7: (**a**) Site map of measurement points 1–1 to 1–3; (**b**) Site map of measurement points 1–4~1–8.

**Figure 8:**
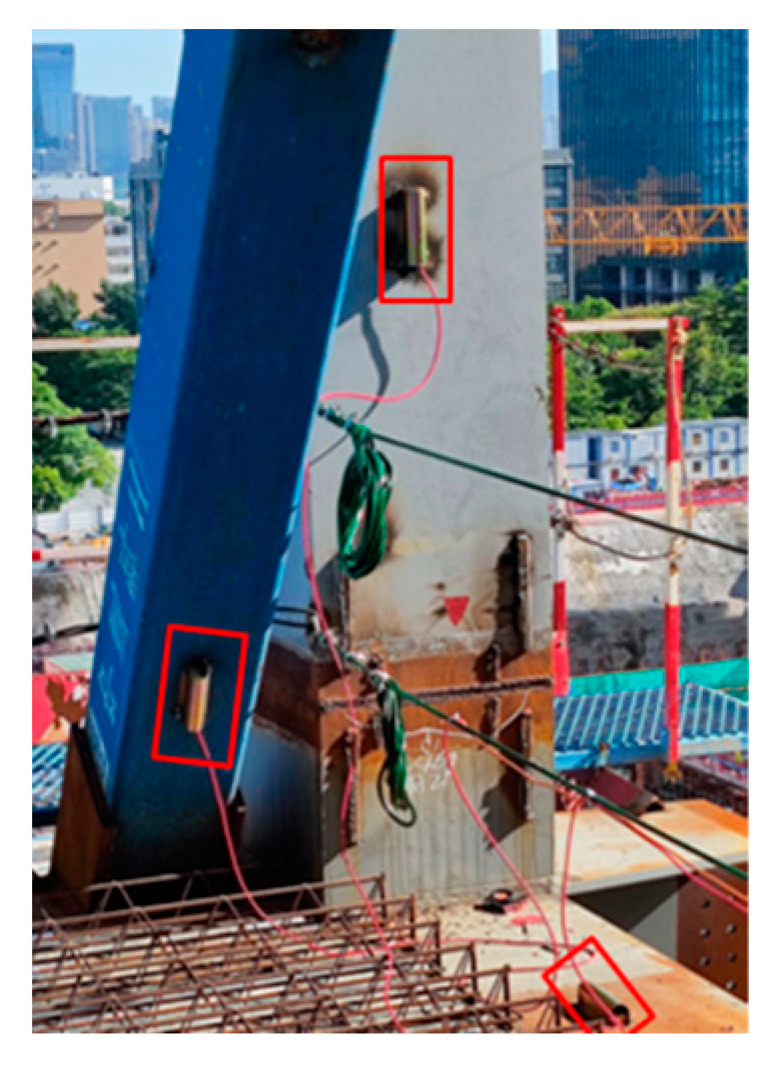
Installation diagram of the sensor (in the red boxes).

The construction monitoring program sets up 88 stress and 20 displacement monitoring points in the steel structure podium and gallery section, wherein stress and strain sensors are embedded with temperature monitoring modules. The monitoring points of the steel structure of the podium and connecting corridor are all located by a steel joist. The statistics of the number and quantity of steel joists, where the measurement points are located, are summarized in Table 1, and the number of steel joists is presented in Figure 3. The stress monitoring points of the GHJ2 and GHJ7 steel joists are illustrated in Figure 6 and Figure 7, and a field installation diagram is presented in Figure 8.

### 3.2. Temperature Measurement

**Figure 9:**
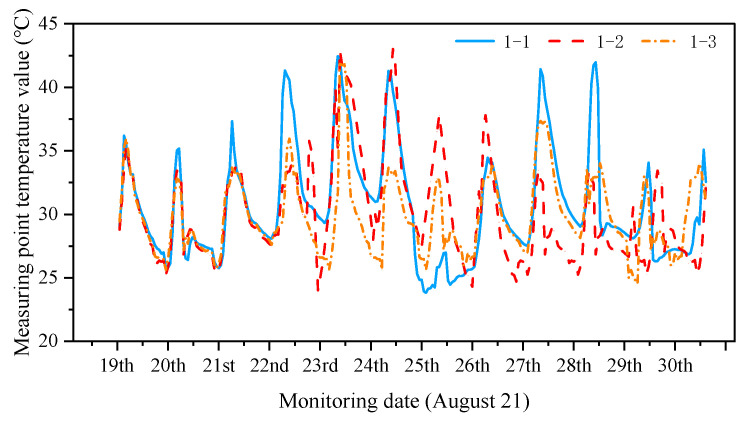
Changes in the temperature monitoring values on the sunny side.

**Figure 10:**
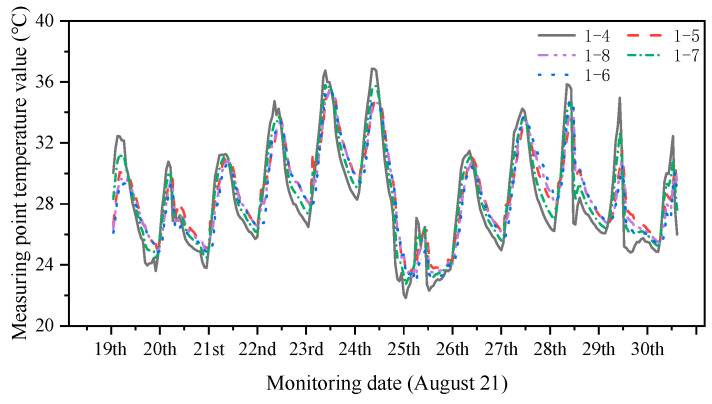
Changes in the temperature monitoring values on the shaded side.

Considering the difference in temperature and degree of solar radiation between the sunny and shaded sides, the on-site temperature monitoring values from 14 August 2021 to 29 August 2021 were selected to plot the change curves of the temperature monitoring values of the sunny and shaded sides (Figure 9 and Figure 10).

Figure 9 and Figure 10 show that the temperature values of the monitoring sites on the sunny side are high in sunny weather, the temperature values of the monitoring sites on the shaded side are low, and the surface temperatures of the structures on the same side change similarly. Five periods of temperature monitoring were selected for the simulation. The temperature monitoring working conditions are listed in Table 2. The temperature was more uniform on the sunny and shaded sides on the rainy day of August 29. For the *i*th construction structure group $J_{i}$, the magnitude of the temperature effects on sunny and shady surfaces under different temperature action conditions is listed in Table 3.

### 3.3. Modeling of Structural Construction in Phases

**Figure 11:**
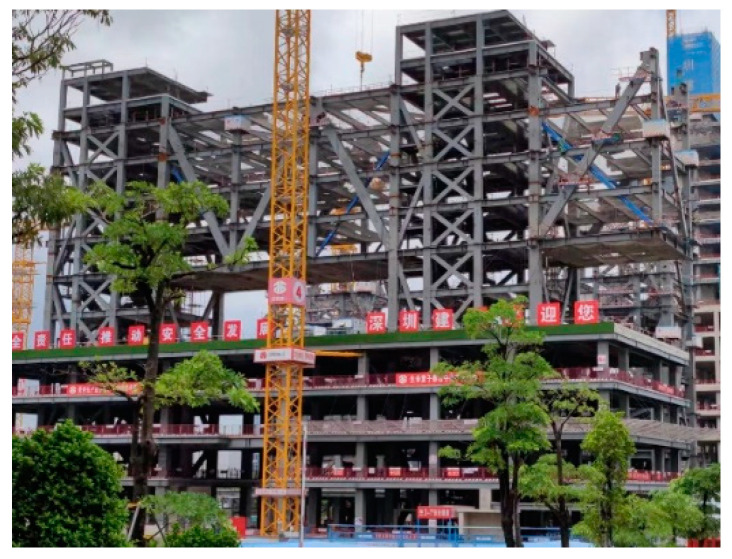
Structural site plan.

**Figure 12:**
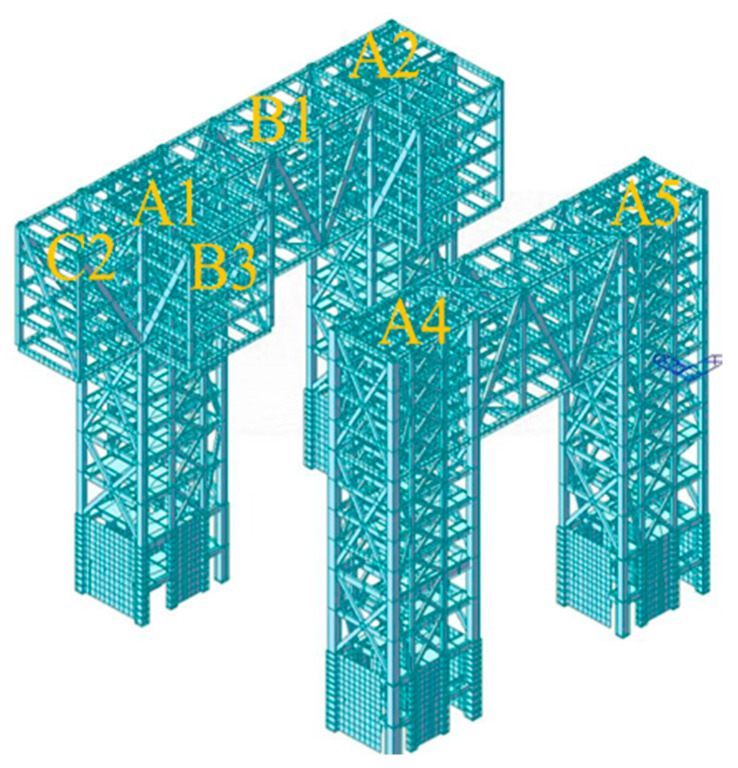
Nanshan Science and Technology Innovation Center A1, A2, and overhanging section.

The research object of this study is a large-span steel structure connecting the corridor part of the three buildings of the Nanshan Science and Technology Innovation Center, which specifically covers the large-span trusses and overhangs between core barrels of A1 and A3, i.e., parts B3 and C2 in Figure 4. The modeling was performed using the MIDAS software. The structural site plan with the created model is shown in Figure 11 and Figure 12. 

### 3.4. Structural Grouping

**Figure 13:**
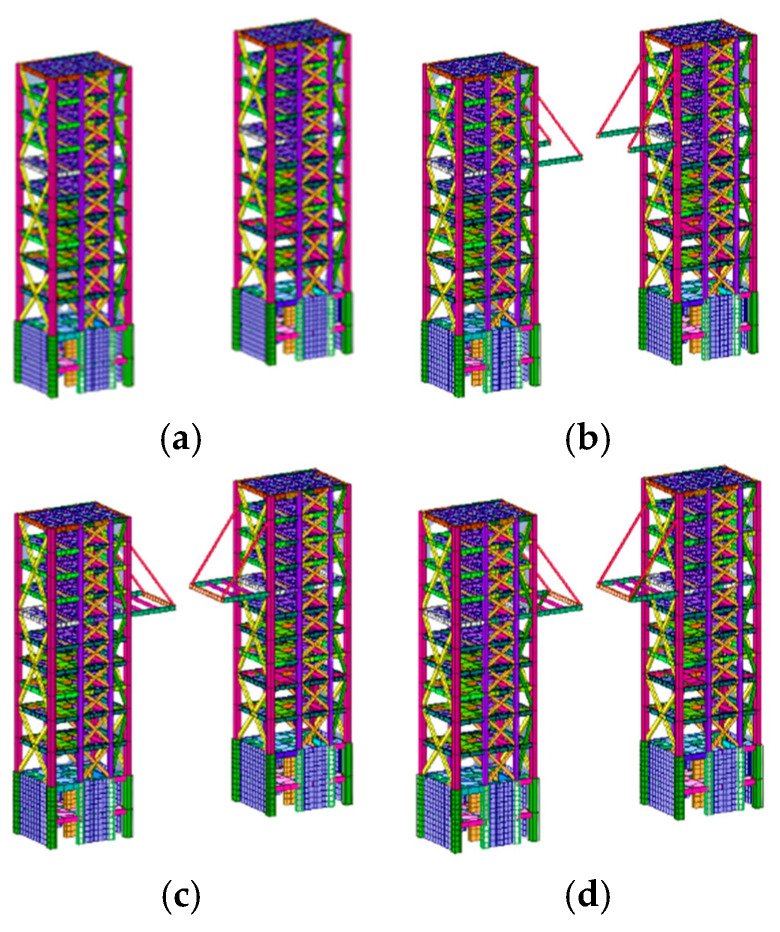
Overview of the construction of some structural groups: (**a**) A1–A2 core; (**b**) structural group 1; (**c**) structural group 2; and (**d**) structural group 3.

A total of 138 construction steps (n = 138) were divided based on the construction steps and sequence of formation of the statically indeterminate structural system, which was divided into 92 construction structure groups (m = 92). Some structural group numbers, corresponding rod locations, and numbers of rods in the structural group are listed in Table 4. As shown in Figure 13a, the construction of area B1 began with the completion of cores A1 and A2. Structural groups 1–44 indicate the construction build processes for area B1, and the first three construction structural groups of this process are shown in Figure 13b–d.

### 3.5. Temperature-Actuated Condition Control

A total of five different temperature-acting conditions were set up to simulate the structural construction process in addition to the temperature-acting condition that considers the initial temperature difference, and four other temperature-acting conditions were set up for comparison (see Table 5). Case k indicates that the construction simulation process considers the initial temperature differences at the time of construction of the statically indeterminate structural temporary stress system. Case b indicates that the construction simulation process does not consider the temperature effect, and cases g and d indicate that the construction simulation process considers a uniform temperature field, where conditions g, d, and c denote the monitored temperature of the sunny side minus the initial temperature of the structure group, the monitored temperature of the back sunny side minus the initial temperature of the structure group, and the one-time application of inhomogeneous temperature action after the structure is constructed and molded, respectively.

## 4. Structural Construction Force Feedback

### 4.1. Displacement Analysis

**Figure 14:**
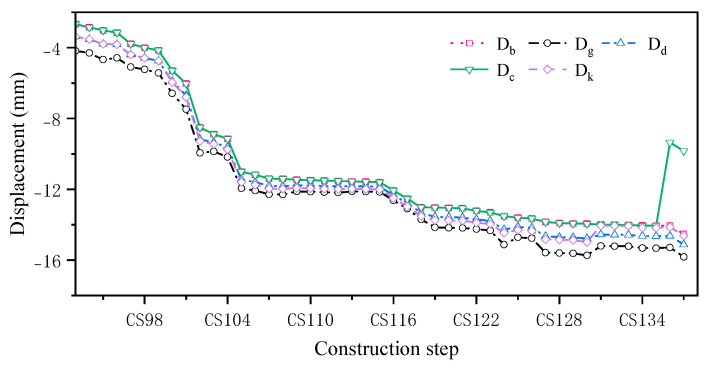
Simulated displacement change curve for monitoring point B3.

The displacement monitoring points in the B3 large-span connecting corridor area in the construction monitoring project of the Nanshan Science and Technology Innovation Center were analyzed. Displacement monitoring data from 21 August 2021 were used to justify the methodology. The simulated displacements at the monitoring points of the B3 span section under the five temperature-acting conditions were compared comprehensively with the construction, and the change curves are shown in Figure 14.

The monitoring point displacements obtained from the construction simulation and measured monitoring point displacements at the site under different temperature operating conditions were compared based on the displacement curves obtained through the construction simulation and comparison of the monitoring displacement data at the construction site (Table 6).

Table 6 indicates that the effect of temperature on the displacement change is not significant at different construction stages and under different temperature action conditions. As the construction proceeds, the difference in cumulative displacements under different temperature conditions is about 1 mm, and the displacement change is small, so the proposed method has little effect on the displacement prediction. The construction simulation method proposed in this paper can be used to reflect the displacement changes in the structure during the construction process.

### 4.2. Stress Analysis

**Figure 15:**
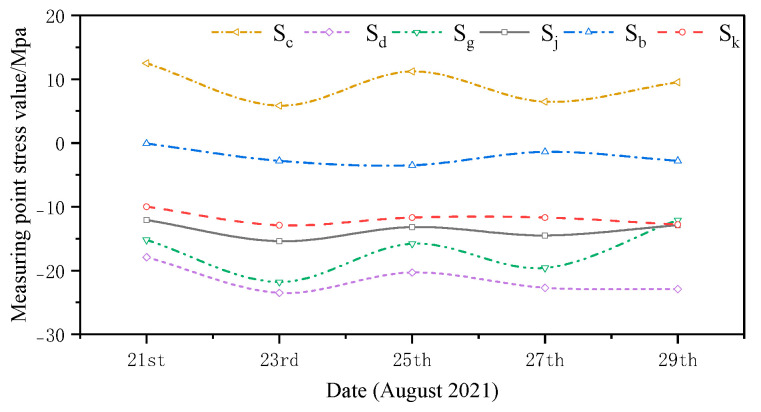
Comparison of stresses at the 1–1 measurement point under the five medium operating conditions.

**Figure 16:**
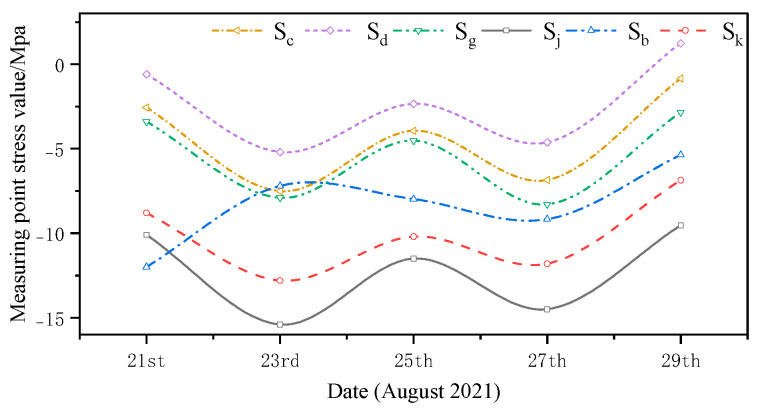
Comparison of stresses at the 1–2 measurement point under the five medium operating conditions.

**Figure 17:**
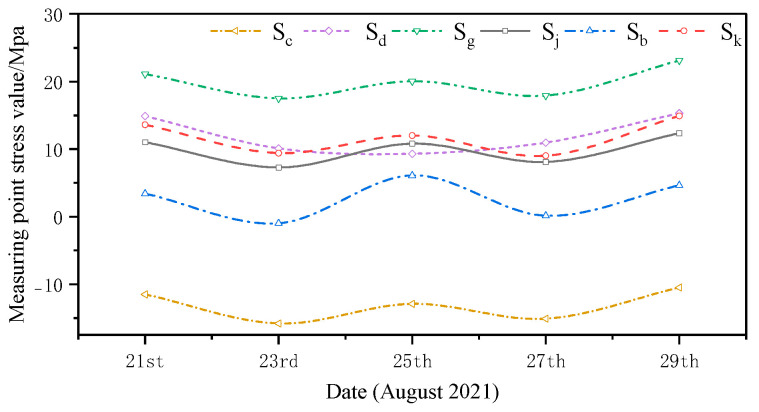
Comparison of stresses at the 1–3 measurement point under the five medium operating conditions.

**Figure 18:**
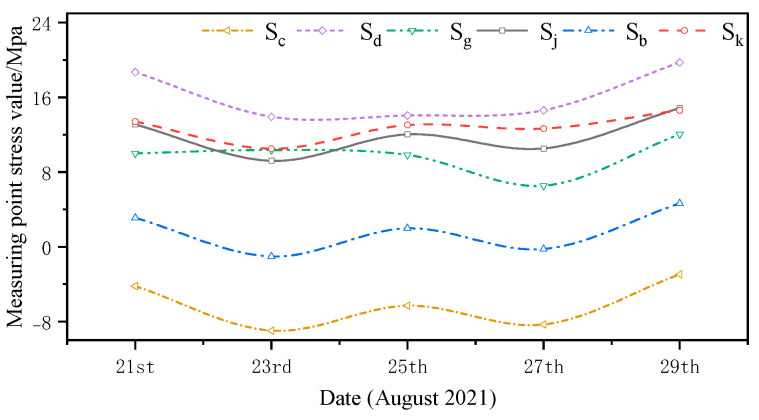
Comparison of stresses at the 1–4 measurement point under the five medium operating conditions.

**Figure 19:**
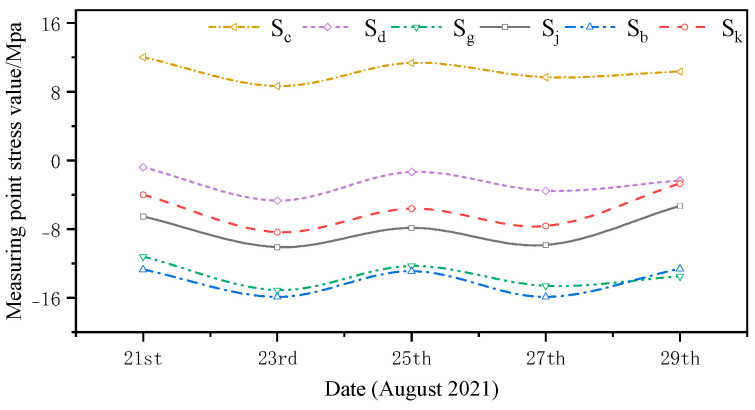
Comparison of stresses at the 1–5 measurement point under five medium operating conditions.

**Figure 20:**
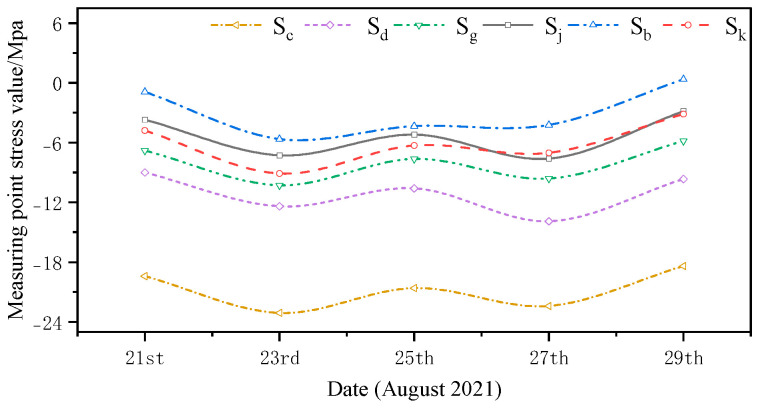
Comparison of stresses at the 1–6 measurement point under five medium operating conditions.

**Figure 21:**
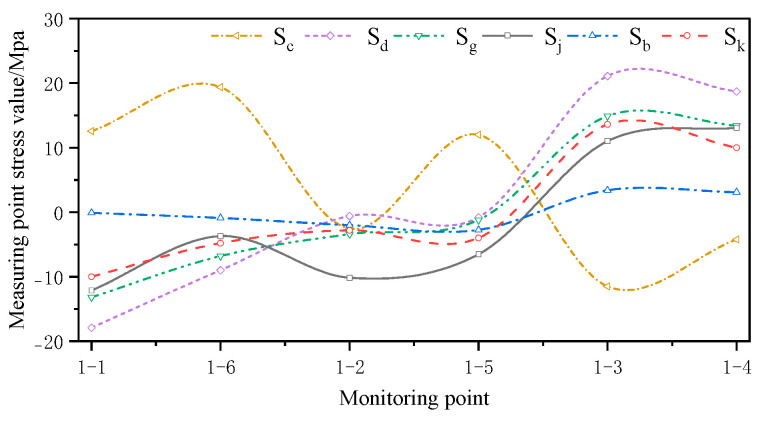
Comparison of simulated and monitored stresses at monitoring points under five working conditions.

The monitored and simulated values of the six stress monitoring point locations in C3 of the overhang end of A1 with the stress monitoring values of $S_{j}$ were compared. A stress comparison of stress monitoring points 1–1 to 1–6 is shown in Figure 15, Figure 16, Figure 17, Figure 18, Figure 19 and Figure 20. The simulated stresses at monitoring points 1–1 to 1–6 under five working conditions on August 23 were compared with the on-site monitoring stress values. The comparisons of the simulated and monitored stresses are listed in Table 7, and the comparison of the simulated and monitored stresses is shown in Figure 21.

In Table 7, $S_{j}$ represents the stress monitoring values, $S_{k}$ represents the stress simulation values from the method proposed by the authors, $S_{b}$, $S_{g}$, $S_{d}$, and $S_{c}$ represent the stress values obtained from other conventional simulation methods, and the error is the error between the stress values obtained from the method suggested in the article and from the conventional simulation methods and the stress monitoring values. Table 7 and Figure 15, Figure 16, Figure 17, Figure 18, Figure 19 and Figure 20 show that the error in temperature stress obtained without considering temperature action is 76.45%, the error in temperature stress obtained by considering uniform warming is 46.33%, the error in temperature stress obtained by considering uniform cooling is 84.41%, and the error in temperature stress obtained by considering a one-time application of inhomogeneous temperature action after completion of the structure construction is 253.5%. Compared with other methods, the temperature stress obtained by the method proposed in this paper is the closest to the measured stress, with an error of ~3 MPa, which can reduce the error by 10–40% compared to other methods.

**Figure 22:**
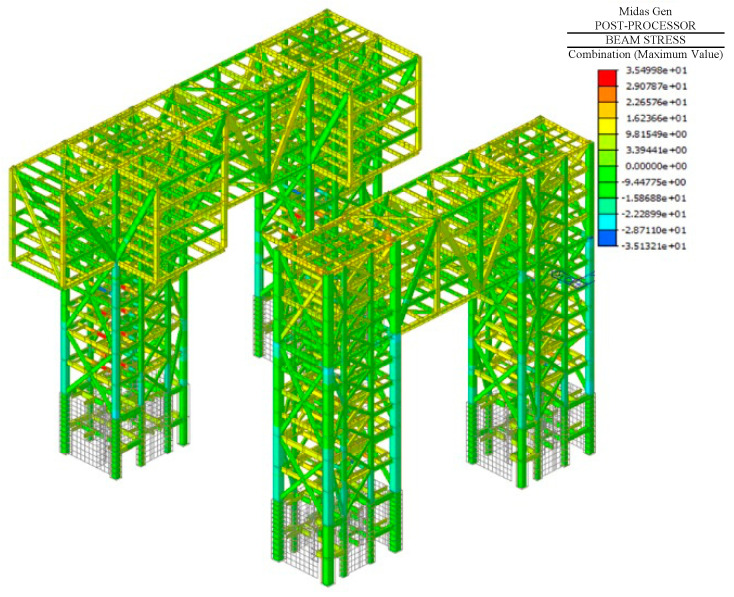
Overall structural stress diagram [MPa].

In conclusion, the method proposed in this study can better reflect the force and deformation conditions of a structure during the construction stage under the action of temperature. According to this method, the stress distribution of the overall structure during the construction phase can be expressed as shown in Figure 22.

In the subsequent construction, this method dynamically calculates the overall structural stresses and deformations in each construction phase and predicts the structural response in the next construction phase, thereby providing guidance for the safe construction of the structure and pre-adjustment of the construction program.

## 5. Conclusions

During the construction of statically indeterminate steel structures, non-uniform temperature action has a large impact on the force condition of the structure. Therefore, based on staged temperature measurement, a feedback method for the statically indeterminate steel structure construction force was proposed in this study. The proposed method obtains the complete stress state of the structure during the construction process, providing a scientific basis for the safe construction of the structure and pre-adjustment of the construction program. The construction simulation method proposed in this study was applied to a construction monitoring project at the Nanshan Science and Technology Innovation Center. Compared with other temperature action calculation methods, the structural force obtained by the method proposed in this study is closer to the actual data measured on-site, and the error is reduced by 10–40%. In this study, we also applied the data extracted from finite element simulation and analysis methods to impose the measurement errors with different signal-to-noise ratios and further demonstrated the quantitative impact of the measured errors on the prediction accuracy of the monitoring methods in the subsequent studies.

## Figures and Tables

**Table 1:** Number and quantity of the steel joist where the measurement point is located.

Steel Truss Number	Strain Temperature Monitoring Points/Unit	Displacement Monitoring Points/pc
GHJ1	0	2
GHJ2	13	2
GHJ3	12	3
GHJ6	10	2
GHJ7	12	2
GHJ8	0	2
GHJ9	3	1
GHJ10	20	2
GHJ11	18	4
Total	88	20

**Table 2:** Temperature monitoring conditions.

Working Condition	Dates	Weather Conditions	Temperature on the Sunny Side/°C	Temperature on the Shady Side/°C
1	August 21	Sunny and cloudless	36	32
2	August 23	Sunny and cloudless	43	37
3	August 25	Sunny and cloudless	36	28
4	August 27	Sunny and cloudless	42	34
5	August 29	Rainy	35	35

**Table 3:** Temperature action values for the sunny and shady sides for each time period.

Working Condition	Dates	Temperature on the Sunny Side/°C	Temperature on the Shady Side/°C
1	August 21	$36-T_{i}$	$32-T_{i}$
2	August 23	$43-T_{i}$	$37-T_{i}$
3	August 25	$36-T_{i}$	$28-T_{i}$
4	August 27	$42-T_{i}$	$34-T_{i}$
5	August 29	$35-T_{i}$	$35-T_{i}$

**Table 4:** Number and quantity of the steel joist where the measurement point is located.

Structure Group Number	Positioning of Rods	Number of Bars/pc
Structural group 1	Bottom and diagonal bracing, seventh floor, area B1	8
Structural group 2	Bottom beam, seventh floor, block B1	6
……	……	
Structural group 45	Bottom secondary beam, seventh floor, area C2	2
……	……	
Structural group 91	Beam on the west side, eleventh floor, area B3	8
Structural group 92	West inclined beam, eleventh floor, block B3	10

**Table 5:** Temperature-activated working conditions.

Serial Number	Working Condition Name	Working Condition	Displacement Expression	Stress Expression
1	Disregarding the temperature effect	b	$D_{b}$	$S_{b}$
2	Maximum uniform temperature rise	g	$D_{g}$	$S_{g}$
3	Minimum uniform temperature rise	d	$D_{d}$	$S_{d}$
4	Temperature applied after molding	c	$D_{c}$	$S_{c}$
5	Considering the initial temperature difference	k	$D_{k}$	$S_{k}$

**Table 6:** Comparison of monitoring point displacements for different temperature-acting conditions.

Working Step	CS91	CS92	Simulated Value of Displacement Change	Monitored Value of Displacement Change	Absolute Error
*D_b_*/mm	−14.06	−14.52	−0.46	−1.00	0.54
*D_g_*/mm	−15.28	−15.82	−0.53	−1.00	0.47
*D_d_*/mm	−14.63	−15.13	−0.50	−1.00	0.50
*D_c_*/mm	−9.35	−9.83	−0.49	−1.00	0.51
*D_k_*/mm	−14.14	−14.62	−0.48	−1.00	0.52

**Table 7:** Comparison of the simulated and monitored stresses at monitoring points for five temperature conditions.

Temperature Conditions	1–1	1–6	1–2	1–5	1–3	1–4	Error/%
*S_j_*/Mpa	−12.1	−3.7	−10.1	−6.5	11.0	13.1	—
*S_k_*/Mpa	−10.0	−4.8	−2.8	−4.0	13.6	10.0	34.2
*S_b_*/Mpa	−0.1	−0.9	−2.0	−2.7	3.4	3.1	76.5
*S_g_*/Mpa	−17.9	−9.0	−0.6	−0.8	21.1	18.7	46.3
*S_d_*/Mpa	−13.2	−6.8	−3.4	−1.2	14.9	13.4	84.4
*S_c_*/Mpa	12.5	19.4	−2.6	12.0	−11.5	−4.2	253.5

## Data Availability

Data are contained within the article.
